# Emotional Atmosphere’s Role on People’s Application of Their Emotional Intelligence in the Fight Against COVID-19

**DOI:** 10.3389/fsoc.2021.581970

**Published:** 2022-02-03

**Authors:** Dinah Katindi Nyamai

**Affiliations:** Educational Administration and Planning (Curriculum Studies), University of Nairobi, Nairobi, Kenya

**Keywords:** Emotional intelligence, emotional atmosphere, application, young people, fight

## Abstract

Emotional intelligence has been associated not only with young people’s academic achievement but also with their ability to deal with harmful emotional states such as anxiety and stress. Limited research, however, has focused on influences of the emotional atmosphere on young people’s application of their emotional intelligence. This article seeks to provoke debates about the role played by accidental lessons arising from learning institutions emotional atmosphere on young people’s ability to apply their emotional intelligence in the fight against life crises such as COVID-19. The target population was 617 young people, aged 15–35 years, and the researcher used Yamane’s sample calculation formula in determining the sample size, which was 243. The researcher used two main data collection tools: a closed-ended questionnaire with 14 items and an interview guide with 10 open-ended questions. The validity of the 14-item questionnaire was determined by experts, whereas its reliability was determined using Cronbach *α*, which gave a reliability coefficient of 0.91. The clarity of the interview guide items was determined by two research experts. The results of both quantitative and qualitative indicated that conducive emotional atmospheres increase people’s mastery of their feelings as well as spur one’s capacity to endure discomforts associated with crises. The implication here is that we ignore the power welded by accidental lessons arising from emotional atmosphere at our peril.

## Introduction

While the COVID-19 pandemic continues to cause anxiety, fear, and stress among many people (Fuchs, 2020), emotional intelligence has an important effect on people’s ability to manage uncertainties that cause worry, fear, and anxiety (Goleman, 1995; Mayuran, 2013). Life challenges cause indescribable anxiety, fear, and worry to almost everybody, but emotional intelligence is strongly related to job-related success and to uncertainty and stress tolerance. Hudson (2018) asserted “emotional intelligence is a set of social and emotional skills that influence how you perceive and understand us, how you express ourselves to others, how you develop and maintain relationships, and, of real interest here, how you cope with challenges.” Those who have high emotional intelligence are aware of their situations, have control over the emotions they experience, and show less stress, which prevents anxiety, fear, or worry from seducing them into negative consequences. This observation echoes Goleman (1998) and Drigas and Papoutsi (2020), who claimed that competencies of emotional intelligence are a prerequisite for good mental health even in stressful situations, and lack of high emotional intelligence skills in unstable environments can lead to questionable consequences.

Scholars such as Rietti (2008) thought emotional intelligence is a fixed trait and therefore unteachable or unlearned, but the consensus view according to Goleman (1995) and Domitrovich et al. (2007) is that emotional intelligence can be learned, and teachers’ social emotions and accidental lessons arising from learning institution’s emotional atmosphere significantly affect students’ emotional outcomes (Jennings et al., 2011). Emotional intelligence competencies mostly develop within natural interpersonal relationships (Shipman and Zeman, 2001), and without emotionally relevant contexts, there is the risk that learning emotional intelligence may be difficult (Tice et al., 2001). Other factors such as genes play a significant role in emotional intelligence development, but lessons arising from learning institutions’ emotional atmosphere account for as much as 90% of all young people’s learning experiences (Alsubaie, 2015, p. 125 and; Massialas and Hurst, 2009). The focus in this research is therefore to provoke debates about the role played by lessons arising from learning institutions’ emotional atmosphere on young people’s ability to apply their emotional intelligence in the fight against life crises such as COVID-19.

Scholars such as Mayuran (2013) and Drigas and Papoutsi (2018) claimed that people require more than cognitive intelligence to be successful in life. One’s “ability to sense, understand, and effectively apply the power and shrewdness of emotions as a source of human energy is the doorway to people’s living a happier life. Goleman (1995) posited that CEOs are usually hired for their business know-how and intellect but dismissed for lack of emotional intelligence. According to Sherlock (2002), people with high emotional are sensitive to other people’s emotions, communicate better, and use active listening. Goleman’s (1995) observation echoes a well-known saying that “magic comes alive when intelligence combines with emotions.” This means effective mastery of one’s emotional competencies can result in greater individual and social success, whereas limited emotional intelligence would lead to a variety of personal and social hitches. According to Drigas and Papoutsi (2020), “people who are proficient in emotion-regulation abilities are generally less likely to collapse under the pressure of real-life stressors. They can keep their cool, handle difficult situations with grace, make other people feel at ease, and are more likely to take precautionary measures for the restoration of their emotional balance and the resolution of their problems.” One’s ability to effectively use his/her emotions to guide his/her thinking and behavior determines one’s success above and beyond intellectual quotient (Hogeveen et al., 2016). Emotions by their very nature leads to one or other impulses to act, which means it can rightly be said that there is “no psychological skill more fundamental than emotional intelligence—one’s ability to manage one’s emotions” (Goleman, 2006, p. 81). Emotional intelligence also matters more than one’s intellectual quotient in maintaining rewarding relationships because it allows people to empathize with other people, be socially aware, and stay sensitively present amid upsetting information without letting the information override their self-control.

Emotional intelligence provides people with a framework via which they can apply cognitive intelligence to emotional responses. One’s overall cognitive intelligence is directly linked with his/her emotional intelligence. Parker et al. (2005) and Kadane, 2018) argued that emotional intelligence predicts people’s mental wellness, and young people who have high emotional intelligence tend to stay in school longer and smoothly transition from high school to higher education because they make better choices in life and are better in terms of collaboration, behavior, and academics. Goleman (2006) and Omid et al. (2016) observed that inappropriate behaviors such as “aggression among young people are often due, in part, to a lack of emotional intelligence, which means young people with high emotional intelligence are not only better in academics and managing their emotions but also in behavior. The implication here is that when one’s emotional capabilities are not in hand—that is, he/she does not have self-awareness, is not able to manage his/her distressing emotions, cannot have empathy, and cannot maintain relationships—no matter how intelligently smart one is, he/she is not going to get very far.

Stress is a useful human response because without it, people would not react correctly to threats or perform faster in some tasks, excess stress can exacerbate serious health problems, and people need emotional intelligence to connect with their feelings and turn detrimental stress, anger, and fear into productive activities (Kalpan and Sadock, 1998). According to Segal et al. (2019), “it is not the smartest people who are the most successful in life because there are academically brilliant people who are socially inept and unsuccessful. This means people’s cognitive intelligence is not sufficient on its own for one to achieve success in life, and if it were so, we would not have some young people performing very highly academically and even rated as the best students in school, but are unable to handle stress, fear, and psychological trauma, and as a result, they abuse drugs, and worse still, some get radicalized. Houston (2020, p. 1) argued that emotional intelligence improves people’s relationships and facilitates their ability to read and navigate a plethora of stressful situations, which closely links emotional intelligence to resilience.

The concept emotional intelligence was coined by Salovey and Mayer (1990), but over the years, it has evolved from its inception as “social intelligence” in the early 1930s, to “emotional strength” in the mid-20th century to its current terminology, “emotional intelligence.” This article examines consequences of accidental lessons arising from learning contexts’ emotional atmosphere on young people’s ability to apply their emotional intelligence in effectively managing life challenges such as COVID-19 pandemic. The researcher discusses the phenomenon along Mayer and Salovey’s (1997) four-branch definition of emotional intelligence: (1) perceiving and identifying motions, (2) using emotions to facilitate thought, (3) understanding emotions, and (4) managing emotions. As noted by Segal et al. (2019), a positive emotional atmosphere (hidden curriculum) promotes people’s application of their emotional intelligence—their “capability to recognize their own emotions and those of others, discern between different feelings and label them appropriately, and use the emotional information to guide their thinking and behavior.” This means creating positive emotional atmospheres—naturally mapping people’s IQ with social–emotional skills such as motivation, impulse control, coping mechanisms, and the ability to delay gratification—is likely to reduce unnecessary fear, anxiety, and stress and effectively help many young people, if not all of them, overcome many life challenges. On the contrary, inability to manage anxiety, stress, and frustrations has negative effects not only on young people’s cognitive functioning, but also on their behavior including their capability to manage COVID-19–related challenges.

## Emotional Atmosphere and Application of Emotional Intelligence

The term *curriculum* comes from the Latin word *currere*, meaning to “run a (race) course”—referring to a sequence of steps in teaching and learning specific content (Barani et al., 2011). According Barani et al. (2011), thinking of curriculum as a sequence of learning experiences immediately leads to the conclusion that no one teacher or otherwise can always control every individual student’s learning experience because students learn not only cognitive skills, but also noncognitive ones. The unwritten curriculum is opposed to the official curriculum that aims to develop students’ cognitive skills, and it is seen as having an effect on people’s well-being (Kelly 2009; Kananen, 2020). Accidental lessons arising from the unwritten curriculum are conveyed through body language and other nonverbal cues, and their impact supersedes official lectures in the development of emotional intelligence competencies among young people. The accidental learnings begin long before any official lecture and never stop because the learning environment is part of the educational process, and it continues to surreptitiously teach even when official lectures are over.

Challenges associated with learning often lead to negative consequences especially when learning institutions fail to provide conducive learning atmospheres. But “positive surroundings like having someone smile at you promote one’s self-esteem and well-being as well as puts people in a good mood to empathize with other people” (Gueguen and De Gail, 2003). Educators are therefore better advised if they did not to solely focus on increasing school funding but also create conducive learning emotional atmosphere because children’s acquisition of emotional faculties is a significant milestone in their holistic development (Soltani et al., 2017). Students subconsciously or inadvertently imbibe accidental lessons as the socialization process of education (Jerad, 2006 and Killick, 2016), which are powerful, and indeed power-laden, but gets no consistent, direct scrutiny (Kentli, 2009, p. 83). As noted by Gottman et al. (1997), using everyday emotion-evoking situations as teachable moments is effective in teaching emotional intelligence skills. Parrott and Spackman (2000) also claimed that skills learned in a relevant emotional setting can generalize to other similar emotional contexts.

A lot of cognitive developments come from what children learn through the official curriculum, but no formal curriculum tells the full story. There are other forms of knowledge, such as emotional intelligence, and essential soft skills, including behavior, are accidentally conveyed through the hidden curriculum or the emotional atmosphere. Philip Jackson in 1968 noted that accidental lessons arising from the hidden curriculum socialize young people to effectively adapt to real life. Alsubaie (2015) defined the hidden curriculum or the emotional atmosphere as “all things that are learned during schooling in addition to the official curriculum.” According to Cubukcu (2012) and Alvior (2014), the hidden curriculum can work as “the manipulative curriculum, which is used to manipulate the unwary. In this article, the hidden curriculum or the emotional atmosphere is understood as accidental lessons arising from that which goes on in every learning institution or the way the official curriculum is taught.

Every learning context, including the society, has a hidden curriculum, which communicates ideological messages to the receiver (Foot, 2017; Crossman, 2019). Over the decades, however, most research has focused on the effects of declarative curriculum, which means aims and values of the hidden curriculum, which is taught by whole education systems, have not been given due recognition, yet its lessons are the main gateway to balanced life development. This neglect happens against a backdrop of research upon research that emphasize conducive learning emotional atmosphere as key facilitator of young people’s learning including their application of their emotional intelligence to manage stressful circumstances, which results in practical benefits as it spurs one’s ability to draw on his/her interpersonal skills/intrapersonal skills, self-management skills, conflict resolution skills, and skills to effectively manage relationships (Salovey and Mayer, 1990).

### Interpersonal and Intrapersonal Skills

The terms *interpersonal* and *intrapersonal* are often used interchangeably, but there is a distinct difference between the two terms. Whereas intrapersonal skills involve emotions including thoughts and feelings within an individual—not visible to other people around the person, interpersonal skills are readily visible to other people. As noted by Shek (2010), “intrapersonal skills go a long way in producing good interpersonal relationships—the ability to interact well with other people, which means interpersonal relationship skills are an important strategy to facilitate holistic development of young people.” This observation is in line with Bar-On and Parker (2000), who argued that “interpersonal skills consists of empathy, understanding of feelings, ability to create and sustain fulfilling relationships, and social responsibility, while intrapersonal skills involve assertiveness, realization of one’s potential in terms of self-directedness, self-controlled thinking, cognitive restructuring (e.g., identifying and stopping maladaptive thinking), accurate processing of social information, and perspective taking.” According to Mayer and Salovey (1997), interpersonal involving verbal and nonverbal behaviors, such as one’s ability to interpret facial expressions and maintain eye contact, appropriate adjustment of one’s tone, and volume of voice, as well as correct response to physical social cues, can lead to more refined and successful interactions. In simple terms, intrapersonal skills are one’s ability to differentiate between positive and negative feelings in oneself and react appropriately, which means a conducive emotional atmosphere is particularly important especially during crises times such as the COVID-19 pandemic, which has exposed thousands of people to negative consequences such as a lack of social support.

### Self-management and Self-efficacy

Self-efficacy has been identified by researchers such as Goleman (1995) and Chandra (2020) as key skills in handling stressful situations such as current COVID-19 pandemic in ways that result in positive outcomes in specific tasks. A lack of emotional intelligence skills such as ability to resist or delay impulsive reactions to stressful situations, would result in failure that can negatively affect an individual’s future. The concept of self-management has to do with self-regulation, which positively influences people’s performance (Mayer and Salovey, 1997). This means young people who are able to cope with stress associated with academic challenges perform better than those who do not have the capacity to manage their stress because they are able to recognize the difficulties and avoid becoming engrossed to the situation or being flooded by negative emotions. Van der Kolk (1994) argued that emotional intelligence enables one’s brain to cope with stressful situations. The need therefore to determine the role played by accidental lessons arising from the emotional atmosphere in people’s application of their emotional intelligence during and after COVID-19 crisis cannot be overemphasized because people’s emotions—either positive or negative—arise from one’s response to either internal or external happening.

### Self-awareness and Social Skills

As already alluded to, self-awareness and social skills are matters of emotional intelligence whose role in people’s success is undeniable. Research conducted over a million people by Talent Smart cited by Bradberry (2016) suggested that “people who can discern other people’s emotions and empathize with their perspective” are more successful at their workplace relationships because they can effectively resolve conflicts or even prevent the conflict before they start. According to Mayer and Salovey (1997), people with higher levels of emotional intelligence are better at negotiations. This means emotional intelligence can help people in having stronger internal motivators, which can in turn increase their self-confidence as well as improve their ability to focus on more beneficial issues. Emotional intelligence also helps people understand what motivates other people, which in turn helps them in building stronger relationship bonds. This means emotionally shrewd people, both young and old, can build stronger teams by strategically utilizing the emotional diversity of other people to benefit their teams. This article therefore discussed influences of learning contexts’ emotional atmosphere (hidden curriculum) on the holistic development of young people’s emotional intelligence so as to suggest effective viable ways of nurturing high emotional intelligence, creativity, and self-regulation among our present and future leaders. The research was guided by the following three research objectives:

(1) To examine how social–emotional atmosphere affect young people’s interpersonal and intrapersonal skills in the fight against COVID-19
(2) To determine how social–emotional atmosphere affects young peoples’ self-management skills in the fight against COVID-19
(3) To investigate how social–emotional atmosphere affects young people’s self-awareness and social skills in the fight against COVID-19

## Methods

The method of the study was mixed methods. The researcher used both qualitative and quantitative data collection methods in collecting and analyzing data on the phenomenon because combining various types of data strengthens the validity of research findings. While quantitative data summarize and rationalize collected data into useful information, Patton (2015) observed that qualitative data provide rich information and facilitate a depth of understanding not possible through quantitative data alone. Patton’s argument is in line with other researchers who suggest culture should be observed, more than measured. Hence, in order to minimize inadequacies in either research approach, as suggested by Creswell and Clark (2011) and Hubbard (2010), the researcher used a closed-ended questionnaire, with 14 items among 243 young people aged 15–35 years in areas in Nairobi City County considered as a hot spot of COVID-19 and an interview guide with 12 open-ended items among 10 key informants.

### Research Respondents

The research population consisted of 617 young people from one area in Nairobi that was considered coronavirus hot spot. The sample size in the quantitative phase was determined using Yamane’s sample calculation formula given by *n* = N/(1 + Ne^2^), where n is corrected sample size; N, population size; *e*, margin of error (MoE), *e* = 0.05. Thus, from a target population of 617 (1+617 (0.05^2^) = 617/2.54 = 2,242.91–243) young people, who were selected through stratified random sampling. The researcher divided the young people into two strata according to their gender and proportionately selected subgroups: 392/617* x *243 = 154.38–154; 225/617* x *243 = 88.61–89. According to Creswell and Clark (2011), stratified sampling produce are more inclusive because they incorporate subgroups of small populations, which researchers are likely to leave out if they use other sampling procedures. The proportionally selected samples from male and female young people drawn from each gender are summarized in Table 1:

**TABLE 1:** Sample summary.

	Population	Sample
Male	392	154
Female	225	89
Total	617	243

For the in-depth interviews, the researcher purposively selected 10 key informants—young people who the researcher perceived has specialist knowledge—and had extensive valuable information about what was happening than other youth. According to Welman and Kruger, 1999), “purposive sampling is the most important kind of nonprobability sampling because it helps researchers identify primary participants who have experienced the phenomenon being researched.” The researcher handpicked five female and five male youth leaders who were leading a large youth group, at least 10 young people, encouraging them to follow ministry of health guidelines in the fight against COVID-19.

### Research Tools

The questionnaire used to collect data for this research consisted of 14 closed-ended items with 5-point Likert scales: strongly agree, agree, not sure, disagree, and strongly disagree. The validity of the questionnaire was confirmed by five experts, whereas its reliability was determined through a pilot test among 24 young people in similar conditions but not part of the research sample. The researcher used Cronbach *α* to determine the internal consistency of items under each research questions, which on average gave a desirable α coefficient of 0.91). The interview guide’s clarity was determined by 2 research experts and through pilot testing with 5 students who were not part of the research sample. The researcher personally distributed the 243 questionnaires and after a week she collected the filled questionnaires. All the questionnaires distributed were correctly filled and returned. The face-to-face interviews with each of the key informant took about 40 min and the researcher audiotaped informants’ responses and transcribed each audio tape record verbatim.

### Data Analysis

The quantitative data were analyzed using descriptive and Pearson correlation statistics in the SPSS software program version 25. The descriptive statistics were used to determine frequency of respondents on an item in the questionnaire based on the 5-point Likert scales: strongly agree, agree, not sure, disagree, and strongly disagree, which the researcher compressed into three: agree, not sure, and disagree for easy analysis. The Pearson correlation coefficients were used to test the strength of the relationship between young people’s social–emotional atmosphere and their motivation or willingness to adhere to health guidelines in the fight against COVID-19, according to the three research hypotheses:

H0_1_: There is no significant relationship between learning social emotional atmosphere and students’ application of their interpersonal and intrapersonal skills in the fight against COVID-19.
H0_2:_ There is no significant relationship between learning social emotional atmosphere and students’ application of their self-management skills in the fight against COVID-19.
H0_3_: There is no significant relationship between learning social emotional atmosphere and students’ application of their self-awareness and social skills in the fight against COVID-19.

The grounded theory analytical approach was utilized in analyzing the textual data because it was perceived as offering a more neutral view of informants’ experiences with the phenomenon and its influences on their behavior. The researcher read textual data from each key informant several times. She identified recurring themes, carefully coded each emerging theme with similar keywords and phrases, hierarchically clustered codes into key ideas, and then developed thematic categories through relationship identification.

### Discussions on Research Results

The descriptive statistics on research questions 1 concerning how the emotional atmospheres affects their interpersonal and intrapersonal skills in the fight against COVID-19 indicated that 82% of the respondents agreed that fear of stigmatization make them defensive to criticism during these stressful times of COVID-19. Ninety-one percent of the respondents agreed that friendly surroundings have kept them calm under the pressure of COVID-19—meaning their mood is not negatively affected. Fifty-five percent (55%) of the respondents agreed that their friends’ way of life empowers them to effectively handle setbacks related to COVID-19, and as a result, they have achieved almost all their goals. However, a good number, 45%, of the respondents disagreed that their friends’ way of life empowers them to effectively handle setbacks related to COVID-19, and as a result, they have achieved almost all their goals. Ninety-one percent (91%) of the respondents agreed that mistrust of the government increases anxiety, stress, anger, and fear of COVID-19 among people. These findings concur with Goleman (1998) and Drigas and Papoutsi (2020), who claimed that competencies of emotional intelligence are a prerequisite for good mental health even in stressful situations, and lack of high emotional intelligence skills in unstable environments can lead to questionable consequences.

The researcher conducted Pearson correlation analysis to test the null hypothesis (**1**) there is no significant relationship between learning contexts’ emotional atmospheres and students’ application of their interpersonal and intrapersonal skills in the fight against COVID-19.

According to Table 2, a *p* = 0.012 was obtained in the level of confidence of 95%, which was less than the significance level of *α* = 0.05, which led to the rejection of the null hypothesis arguing that there is a significant relationship between learning emotional atmospheres and students’ application of their interpersonal and intrapersonal skills in the fight against COVID-19.

**TABLE 2:** Relationship between emotional atmospheres and students’ application of their interpersonal and intrapersonal skills in the fight against COVID-19

Statistical index	N	R	Df	sig
Variables				
	243	0.504	243	0.014

The descriptive statistics on research questions 2 concerning how the emotional atmosphere affects their self-management skills in the fight against COVID-19 revealed that 84.8% of the respondents agreed that surrounding circumstances negatively affected their confidence to remind people about washing their hands, wearing their masks, or keeping social distance to reduce the spread of coronavirus, whereas 15.2% disagreed that surrounding circumstances negatively affects their confidence to remind people about washing their hands, wearing their masks, or keeping social distance to reduce the spread of coronavirus. One hundred percent of the respondents agreed that the positive outlook toward life as they see in people around them gives them an optimistic view of life, which in turn gives them the ability to manage despair. Approximately 65% of the respondents agreed that everybody around them maintains a sense of humor even amid COVID-19 storm, which motivates them to manage their stress and anxiety whereas the rest, 34.6%, of the respondents were not sure if everybody around them maintains a sense of humor amid COVID-19 storm and that motivates them to manage their stress and anxiety. Sixty-six percent (66%) of the respondents disagreed that because everybody is trying to see things from other people’s perspective, nobody is struggling to follow health ministry guidelines, whereas 34% agreed that because everybody is trying to see things from another’s perspective, nobody is struggling to follow health ministry guidelines. These findings echo Tice et al. (2001), who claimed that without emotionally relevant contexts there is the risk that learning emotional intelligence may be difficulty.

The researcher conducted Pearson correlation analysis to test null hypothesis 2 generated from objective 2; there is no significant relationship between learning contexts’ emotional atmospheres and students’ application of their self-management skills in the fight against COVID-19.

According to Table 3, a *p* = 0.024 was obtained in the level of confidence of 95%, which was less than the significance level of *α* = 0.05 which led to the rejection of the null hypothesis arguing that there is a significant relationship between learning contexts’ emotional atmospheres and students’ application of their self-management skills in the fight against COVID-19.

**TABLE 3:** The social organizational structure learning contexts’ emotional atmospheres and students’ application of their self-management skills in the fight against COVID-19

Statistical index	N	R	Df	sig
Variables				
	243	0.594	243	0.024

The descriptive statistics on research questions 3 concerning how the emotional atmosphere affects people’s self-awareness and social skills, 82.9% of the respondents agreed that when people graciously tell them how their behavior affects them, they happily keep social distance, wash hands, sanitize, and put on a mask to curb the spread of coronavirus, whereas 17.1% indicated that the way they are told how to manage spread of COVID-19 did not matter. Ninety-five percent (95%) of the respondents agreed that they air their opinions about how to overcome COVID-19 because people around them do not take offense easily while 5% indicated that even if people around them did not take offense, they would still be sacred to air their opinions. Hundred percent (100%) of the respondents agreed that the loving environment around them enables them to listen to self and others without jumping to judgment, which has kept them glued to one another during the COVID-19 pandemic. Eighty-one percent (81%) of the respondents agreed that hostile surroundings make it hard for many people to admit they have COVID-19 symptoms, whereas 19% indicated the environment is not to blame for anything. More than 80% of the respondents indicated that conducive emotional atmospheres, such as trust and a sense of belonging, encourage people to take risks in facing challenges. Fifty-six percent (56%) of the respondents disagreed with the idea that everybody in their surroundings was trying to follow health ministry guidelines, which means there exists negative environmental factors that can derail young people’s energy to fight against COVID-19, such as lack of clarity on issues, lack of motivation from some law makers (especially when they behave like only the common people are vulnerable to coronavirus). These findings concur with the observations of Tice et al. (2001) that without emotionally conducive social atmospheres, there is the risk that acquiring emotional intelligence skills may be difficult.

The researcher conducted Pearson correlation analysis to test null hypothesis 3: there is no significant relationship between learning contexts’ emotional atmospheres and students’ application of their self-awareness and social skills in the fight against COVID-19.

According to Table 4, *p* = 0.024 was obtained in the level of confidence of 95%, which was less than the significance level of *α* = 0.05 which led to the rejection of the null hypothesis arguing that there is a significant relationship between learning contexts’ emotional atmospheres and students’ application of their self-awareness and social skills in the fight against COVID-19.

**TABLE 4:** The social organizational structure learning contexts’ emotional atmospheres and students’ application of their self-awareness and social skills in the fight against COVID-19.

Statistical index	N	R	Df	sig
Variables				
	243	0.653	243	0.019

The researcher used grounded analytical approach in analyzing the textual data because it condenses wide-ranging raw textual data into a summary to establish links between the research objectives and the research findings. The results from the textual data generally concurred with the quantitative data. The 10 interviewed participants claimed that there was close link between lessons arising from social–emotional atmosphere and young people’s behavior including their application of their emotional intelligence and must be considered in any teaching learning process. These findings concur with Hudson (2018) assertion that “emotional intelligence is a set of social and emotional skills that influence how you perceive and understand us, how you express ourselves to others, how you develop and maintain relationships and, of real interest here, how you cope with challenges.”

According to participant 05 lamented that some messages arising from the social–emotional atmosphere reinforces the prescribed curriculum, but a great deal of massages arising from the social–emotional atmosphere (hidden curriculum) like the misinformation sensationalized in mass media coverage often contradict it and can ignite a lot of fear among young people. Participant 09 asserted “accidental lessons arising from the emotional atmosphere comprises what is taught outside the prescribed curriculum and goes beyond the specific content of the subject matter, and can be expressed in the school environment, in the classroom climate and its furniture arrangement, in the pedagogical methods, in teacher student interactions, in the student–student interactions, and in many other invisible dynamics.” According to participant 04, “it is important for young people to be discerning about what they see and hear because negative messages whether intended or not can be detrimental to young people’s ability to harness every power in the fight against COVID-19.” Participant 08 added, “We are in a time of massive upheaval with so many things outside our control—including how long the pandemic will last, how other people behave, and what is going to happen in our communities. We are living in tough times coupled with challenging surroundings, which can negatively affect how young people react to the pandemic.” This observation was echoed by participant 06, who pointed out that many people, especially the youth, are responding to the sensationalistic coverage by endless Internet search for answers. Participant 01 advised that when “one feels like getting caught up in fear, anxiety, and grief over what might happen, he/she should try to shift his/her focus to things he/she can control. For instance, one may not control how severe the coronavirus outbreak is in his/her community, but by taking key steps to reduce their own personal risk, although washing hands frequently with soap and water or sanitizing, avoiding touching their face, and staying home as much as possible is within their power.”

According to participant 03, people’s anxious minds can easily overestimate the actual threat during uncertain circumstances such as current coronavirus pandemic and even underestimate the possibility of them catching the disease especially when nonverbal messages conveyed through mass media—politicians violating rules of avoiding crowded places as though only nonpoliticians can catch the virus, contradict verbal communication. This response concurs with quantitative data, which revealed a significant relationship between people’s application of their emotional intelligence to situations and their cultural reminiscences. For example, participant 05 claimed that “within societies—people’s neighborhoods, in which they share their lives with other people–they learn how to feel in certain situations—even in indirect experiences, they understand how they are supposed to react when they feel threatened.” Participant 01 asserted “accidental lessons conveyed through double standard application of the law on those who flout guidelines on curbing the spread of coronavirus makes people doubt the government’s seriousness on the fight against COVID-19.”

Participant 09 complained that corruption was making young people lose trust in the government and the seriousness of the pandemic. This observation was echoed by participants 02 and 10, who suggested that what people at the top do not understand is that people are struggling to survive, and they must show their seriousness in the war by walking the talk specially to encourage young people who are walking for miles to get clean water, and they must pour some to wash their hands. Participant 05 said, “I don’t want to sound radical, but I think community health volunteers should be facilitated to go door to door, checking what people are going through as well as testing people for COVID-19, to emphasize leaders’ seriousness on the war.”

The qualitative data seem to emphasize that the effective change of people’s behavior and attitude is based on trust because it is hard for people to comply with what you are telling them if they do not trust you. This observation is in line with participants 01, 06, and 10, who argued that the emotional atmosphere is a very important factor in socializing people and very crucial in people’s application of their emotional skills in the fight against life crises.

The implication here is that everybody must pay special attention to social surroundings, not only during the COVID-19 pandemic but always because researchers such as Massialas and Hurst (2009); Crossman (2019), and Drew (2021) claim that the hidden curriculum (emotional atmosphere) accounts for close to 90% of all young people’s learning experiences including the way they think and behave. This means the emotional atmosphere is effective on the outcomes of people’s way of behaving. This means a conducive emotional atmosphere is likely to enhance people’s self-awareness, self-confidence, and self-management, which would lead them to be successful in not only fighting pandemics but also in their academics and life in general.

## Conclusion

The three key findings from this research that can be recommended for present and future use include nurturing people’s emotional intelligence as a skill for managing stressful situations which are inevitable in life. The next point is closely linked with the first one as it has to do with enhancing people’s levels of social responsibility, empathy, and interpersonal relationships, which are aspects of emotional intelligence. This means concepts of self-awareness, self-regard, assertiveness, positive interpersonal relations, flexibility, problem-solving skills, stress tolerance, social responsibility, optimism, empathy, and impulse control should be nurtured in young people from an early age in readiness for life challenges. For this reason, ensuring a conducive emotional atmosphere to facilitate people’s application of their emotional intelligence, which ideally lead to understanding one’s fears and anxiety and better management of negative feelings, is not an option. We therefore ignore the power of emotional atmosphere at our peril. There may be no easy formula for responding to crises, but creating a conducive emotional atmosphere has been found to increase people’s mastery of their feelings, as well as spur their capacity to endure discomforts associated with crises.

## Data Availability

The original contributions presented in the study are included in the article/Supplementary material, further inquiries can be directed to the corresponding author.
